# Experimental and numerical investigation of the transient thermal characteristics of twisted nitinol wires in a continuous torsional refrigeration system

**DOI:** 10.1371/journal.pone.0277415

**Published:** 2022-11-17

**Authors:** Haibo Zhao, Kun Wu

**Affiliations:** 1 Department of Energy and Power Engineering, School of Ocean, Yantai University, Yantai, Shandong Province, China; 2 Department of Automobile and Ship Engineering, Yantai Vocational College, Yantai, Shandong Province, China; Southwest Jiaotong University, CHINA

## Abstract

Twistocaloric cooling technology is a novel solid elastocaloric refrigeration to be promising alternatives to conventional compression refrigeration. The transient thermal characteristics of the twistocaloric-effect material and its cooling capacity are critical for this technology. A test rig of the continuous torsional refrigeration system (CTRS) using nitinol wires twisted by a stepping motor was built. The experimental tests show that, the surface temperatures increased as the stepping motor twisted the nitinol wires clockwise, and decreased by untwisting them counterclockwise under the stepping motor speed of 40, 45 and 45rpm. The maximum temperature rise and drop relative to the ambient temperature for the two-twisted-nitinol-wire combinations were 7.1 and 2.6°C, higher than those of 1.4 and 0.6°C for the single nitinol wire, respectively. An optimization program based on a heat conduction model was constructed to attain the potential cooling and heating capacities (PHCCs) of the nitinol wires. Then, PHCCs were introduced into the coupled flow and convective heat transfer model to predict the actual cooling and heating capacities of the CTRS. They were discovered to increase as the number of nitinol wires, the stepping motor speed, and the air velocity. The results can be referred in developing a continuous torsional refrigeration prototype.

## Introduction

Refrigeration and air conditioning consume a substantially large amount of energy, accounting for nearly 20% of the world’s energy consumption. The most common type of refrigeration and air conditioning is vapor compression refrigeration, which uses chlorofluorocarbons, hydrochlorofluorocarbons, and hydrofluorocarbons as refrigerants. Refrigerants such as R22 and R134a have high ozone depletion potential or global warming potential, which means that leakage of them will deplete the ozone layer and worsen the greenhouse effect. On the other hand, environmentally friendly hydrocarbon refrigerants or inorganic compounds, such as R600a and R717, are flammable and toxic Therefore, the search for novel alternative refrigeration methods has become essential.

Solid elastocaloric refrigeration is a new refrigeration technology that could replace conventional vapor compression refrigeration [1–4]. It is based on the elastocaloric effect that occurs when a metal body is subjected to an external load, thereby transforming the austenite phase into the martensite phase and releasing heat, or releasing the load, thereby transforming the martensite phase into the austenite phase and absorbing heat. In contrast to traditional vapor compression refrigeration, solid elastocaloric refrigeration does not use refrigerants, and thus there is no refrigerant leakage contributing to environmental pollution or the greenhouse effect. In recent years, researchers have studied solid refrigeration induced by phase changes in metals, particularly nitinol (a nickel–titanium alloy). Cong et al. [5] discovered a colossal elastocaloric effect from a polycrystalline nickel–manganese–titanium block. Xiao et al. [6] reported the elastocaloric refrigeration effect of several shape memory alloys (SMAs) and discovered that nitinol can undergo rapid phase transformation, is easy to process, and has good fatigue resistance and high refrigeration efficiency. In elastocaloric studies, the external load can be applied by stretching the metal along the axial direction or twisting the metal around its axis, with stretching load being the most commonly used. Li et al. [7] patented a solid refrigerator based on the elastocaloric effect of a ferroelectric thin film. Qian et al. [8–11] proposed an active regenerative elastocaloric cooling system and introduced a single-stage refrigeration cycle prototype made by stretching nitinol wires in a hydraulic-driven prototype. Ossmer et al. [12] reported a refrigeration prototype using solid-solid contact heat exchange for cooling. Schmidt et al. [13] developed a small refrigeration prototype with independent control of several variables that influence the process functions, such as the strain and strain rate. Zhu et al. [14] suggested that material miniaturization, mechanical loading, and the cycle mode should be further optimized and improved when studying the elastocaloric effect of SMAs. Xu et al. [15] established a 2D nonisothermal phase-field martensite model to study the effect of material shape on the superelastic and elastocaloric characteristics of nitinol; their results revealed that the average temperature change rate of a trapezoidal martensite system is lower than that of a rectangular system. Previously, researchers have mostly used tensile and compressive stress to induce SMA deformation, which requires large equipment and refrigeration output that is periodic rather than continuous. Recently, researchers have shown interest in twistocaloric cooling in which a twisting load is applied to the SMA. Wang et al. [16] discovered that replacing tensile elastic refrigeration with torsional refrigeration resulted in large reversible temperature changes. A nitinol (Ni_52.6_Ti_47.4_) wire had a maximum surface temperature change of −17.0°C at an untwisting rate of 50 turns/s. They also found untwisting polyvinylidene difluoride (PVDF) fibers produced a cooling performance of −1.1 K. However, further studies on the twistocaloric heating and cooling effects are still needed, especially for providing continuous output, and so, the following tasks were carried out in the present work.

1. A novel continuous refrigeration test rig was constructed using nitinol wires twisted by using a stepping motor.
2. Experiments were conducted to determine the thermal characteristics of the continuously twisted/untwisted nitinol wires.
3. A heat conduction-based optimization model was built to determine the potential heating and cooling capacities (PHCCs) of the wires.
4. A coupled flow and convective heat transfer (CFCHT) model was built to determine the heating and cooling output of the continuous torsional refrigeration system (CTRS).

## Experimental apparatus

**Fig 1:**
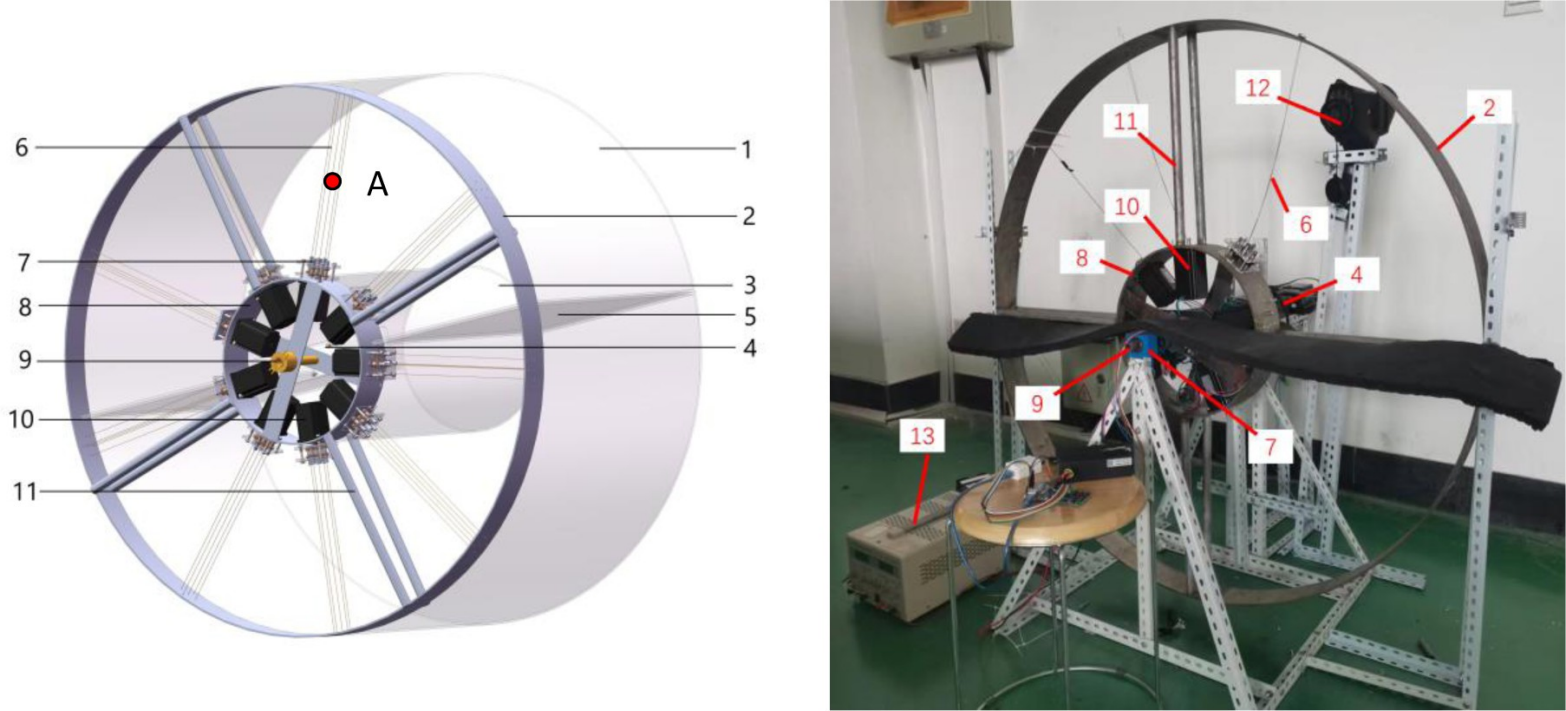
Experimental device of a novel continuous torsional refrigeration system. (a) Schematic Diagram of the Experimental Device. (b) Photo of the Experimental Device. 1—Motor Side External Insulation Layer, 2—Outer Wheel, 3—Insulation Shell, 4—Servo Motor, 5—Heating and Cooling Area Partition, 6—Nitinol Wire, 7—Planetary Gearing, 8—Inner Wheel, 9—Central Shaft, 10—Stepping Motor, 11—Connecting Rod, 12—Infrared Thermal Imager, and 13—DC-regulated Power.

Fig 1 shows the novel experimental device. It is composed of a central shaft, an inner wheel, an outer wheel, stepping motors, a servo motor, nitinol wires, an insulating layer, a conductive slip ring, and planetary gearings. The outer and inner wheels, on which the stepping motors are fixed, are rotated clockwise by the servo motor. One nitinol wire-stepping motor unit is made up of the nitinol wire and the stepping motor. The output shaft of the stepping motor is connected to a nitinol wire. Under the control of the controller, the alloy wire is rotated and twisted by the output shaft of the stepping motor, which makes periodic forward and reverse rotations to load and unload the nitinol wires. The cyclic time is decided by the servo motor.

**Fig 2:**
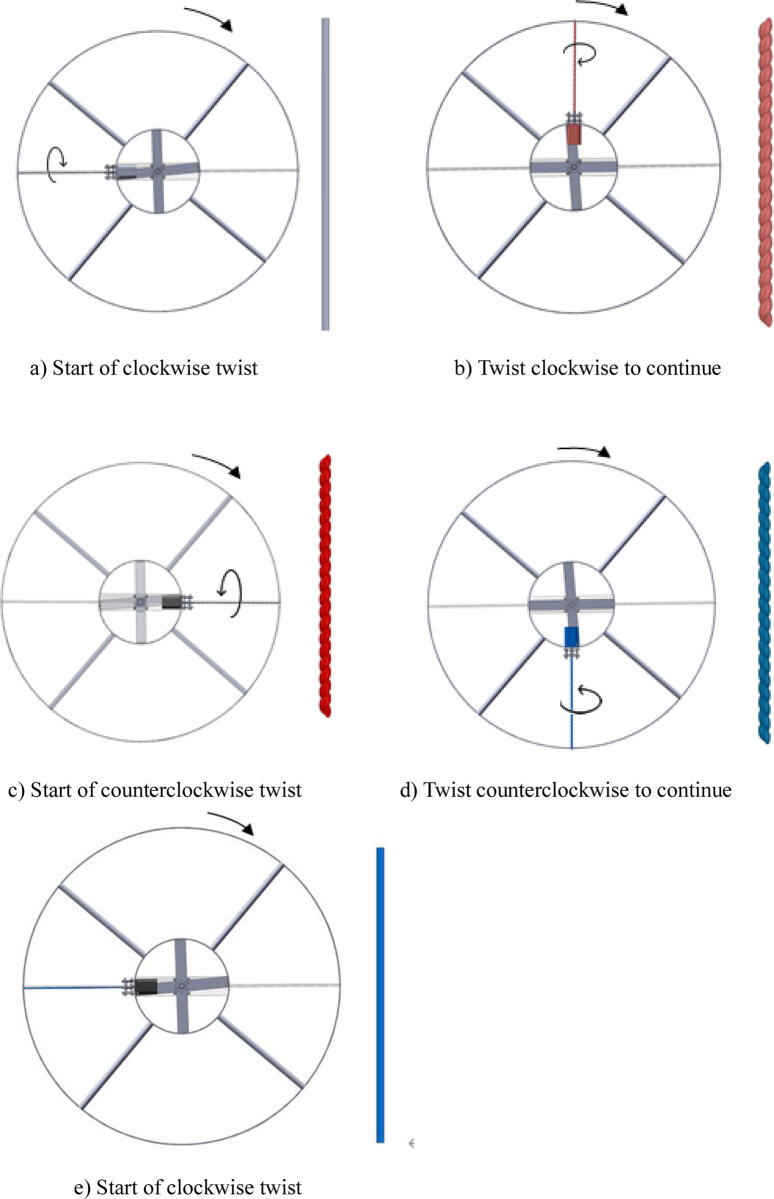
Schematic of the twisted deformation of a nitinol wire during a single operating cycle of the continuous torsional refrigeration system. a) Start of clockwise twist. b) Twist clockwise to continue. c) Start of counterclockwise twist. d) Twist counterclockwise to continue. e) Start of clockwise twist.

**Fig 3:**
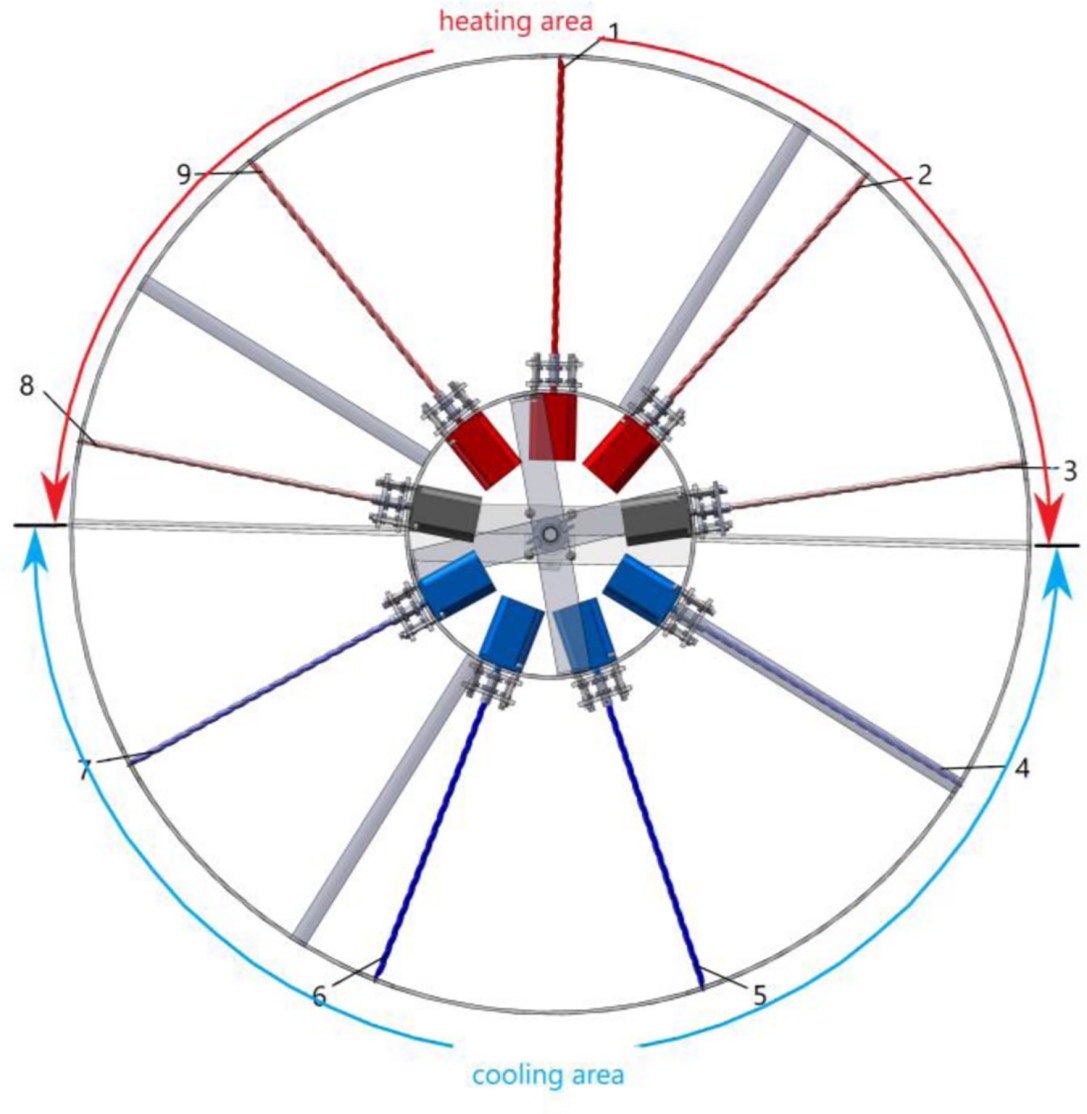
Heating and cooling zone diagram of the continuous torsional refrigeration system.

As shown in Fig 2, when the servo motor rotates, the stepping motor rotates with the inner wheel. When the stepping motor enters the upper half-circle (Fig 2A–2C), it loads the nitinol wire, which twists and releases heat as it transitions from the austenite phase to the martensite phase; the corresponding upper-half zone is the heating zone. When the stepping motor enters the lower half-circle (Fig 2C–2E), it begins a reverse rotation to unload the nitinol wire, causing the wire to absorb heat as it transitions from the martensite phase to the austenite phase; the lower-half zone is the cooling zone. In this manner, the servo motor drives the outer wheel, inner wheel, and stepping motors to rotate in a complete circle. A controller controls the rotational direction of each stepping motor, allowing it to alternate between forward and reverse rotations when it enters the upper and lower half-zones, respectively, as shown in Fig 3. In this manner, each alloy wire completely absorbs and releases heat in the heating and cooling zones, respectively. The two zones of the device can both produce continuous heating and cooling outputs at the same time. The continuous heating and cooling experimental device can be considered a module. Several modules can be connected in series to form a large-scale continuous refrigeration experimental system.

Specifications of the CTRS are listed in Table 1. The wires connected to the stepping motor and the outer wheel of the test rig can be a single nitinol wire or a combination of two nitinol wires twisted clockwise. The slip ring ensures power supply and signal transmission while preventing power supply lines and signal lines from intertwining during the overall rotation process.

**Table 1:** Specifications of the CTRS.

Equipment	Specifications
nitinol wire	a nickel content of 56%, a titanium content of 34%
servo motor	Tode SDGA-01C11AB type DC brushless motor; rated power: 100W; Torque: 12 Nm; rated current: 5.5 A. Driver: TSDA-C11A type, speed accuracy ±2rpm; tracking error: ±1pusle; positioning accuracy: 1/10000
stepping motor	Rtelligent 57CM23 type, rated current: 3.2 A; torque: 2.2 Nm; step angle: 1.8°; DM542-type digital controller, 24 VDC, and a bidirectional pulse output
slip ring	SenRing H2586-2410 type, Voltage range: 0-600V; Current range: 0-10A/route; Aperture range: 25mm; Maximum Route: 24; maximum speed:250rpm
infrared thermal imager	Testo 8852, High quality wide-angle lens 32° × 23. Detector type fpa320 × 240 pixels, thermal sensitivity (NETD) < 60mk, 2GB SD card, minimum focal distance 20cm
DC-regulated power	Mingwei PSP-600 type, output: 24V DC voltage, 25A rated current, 600W rated power output; Input: Voltage range 88-264VAC, frequency range 47-63HZ; 3.4A AC current,
microcomputer controller	STC89C52 series
LCD	12864 LCD, driven by 5V DC voltage, 16 × 16 dot matrix, 128 characters (8× 16 dot matrix), 64 × 256 dot matrix display RAM. Serial communication with external CPU.
standard thermometer	Range: 0–25°C,25–50°C; Accuracy:0.05°C

## Simulation model

### Heat conduction model

#### Governing equation

The heat conduction model of a nitinol wire was built. Nitinol wires are considered infinite cylinders because their diameter is much shorter than their length. Heat is conducted mainly along the radial direction. Therefore, the governing equation is

$$\rho c\frac{\partial t}{\partial \tau }=\frac{1}{r}\frac{\partial }{\partial r}\left(\lambda r\frac{\partial t}{\partial r}\right)+\frac{1}{r^{2}}\frac{\partial }{\partial \varphi }\left(\lambda \frac{\partial t}{\partial \varphi }\right)+\frac{\partial }{\partial z}\left(\lambda \frac{\partial t}{\partial z}\right)+Q_{v},$$ (1)

where *ρ*, *c*, and *λ* are the density, specific heat capacity, and conductivity of the wire, respectively; *t* is the temperature; τ is the time; *Q*_*v*_ is the amount of heat produced or absorbed per unit volume during the torsion process; and *r*, *φ*, and *z* are the cylindrical coordinates.

### Boundary conditions and equations

The initial temperature was ambient temperature. The wire was involved in the heat transfer between ambient air and radiation. The surface heat transfer boundary equation can be written as

$$q_{r}=\varepsilon \sigma (T_{}^{4}-T_{a}^{4}),$$ (2)

where *q*_*r*_ is the radiant heat flux density, *ε* is the emissivity of the material, *σ* is the radiation constant of the black body, *T* is the surface temperature of the emitting surface, and *T*_*a*_ is the ambient temperature.

The heat transfer boundary equation of the wire convection heat flux is expressed as

$$q_{c}=h(T-T_{a}),$$ (3)

where *q*_*c*_ is the convective heat flux density, *h* is the convection heat transfer coefficient, which is calculated using natural convection correlations, where the air velocity is coupled with the airflow model [17].

### Optimal calculation

The *Q*_*v*_ during the torsional process in the governing equation is unknown. According to the temperature evolution of the wires in the nitinol–stepping motor unit, *Q*_*v*_ in the governing equation is described as *Q*_*vd*_ and *Q*_*va*_ during loading and unloading, respectively.

Heat disposal by the loaded wire is expressed as

$$Q_{vd}=Q_{d0}e^{-k_{1}\tau },$$ (4)

Heat absorption by the unloaded wire is expressed as

$$Q_{va}=Q_{a0}e^{-k_{2}\tau },$$ (5)

where *Q*_*d0*_, *Q*_*a0*_, *k*_*1*_, and *k*_*2*_ are constants.

**Fig 4:**
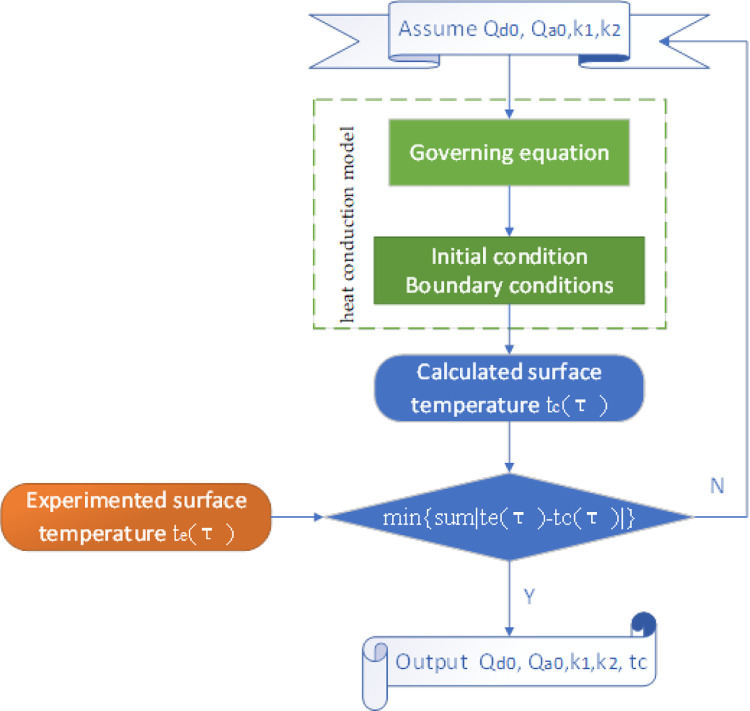
Flow chart of the least-square optimization calculation program.

Therefore, based on the above simulation program, we built an optimization model (Fig 4) that took the minimum difference between the simulated and experimental values of the surface temperature of the nitinol wire as the optimization objective, and took the parameters in Eqs (4–5) as the optimization variables to obtain the value of the constants. The multiparameter optimization was solved using the moving asymptote optimization algorithm (MAOA) [18]. The MAOA is an efficient algorithm for solving nonlinear problems. Based on the linear expansion of Taylor series, the algorithm obtains a series of convex subproblems to replace the original problem, then solves the subproblem with duality method, and uses iterative method to continuously approach the solution of the original problem by the solution of a series of moving asymptotic subproblems. In each iteration, the original problem is simplified, a strictly convex separable approximate subproblem is generated, and the optimal solution of the original problem is obtained step by step.

### The CFCHT model

**Fig 5:**
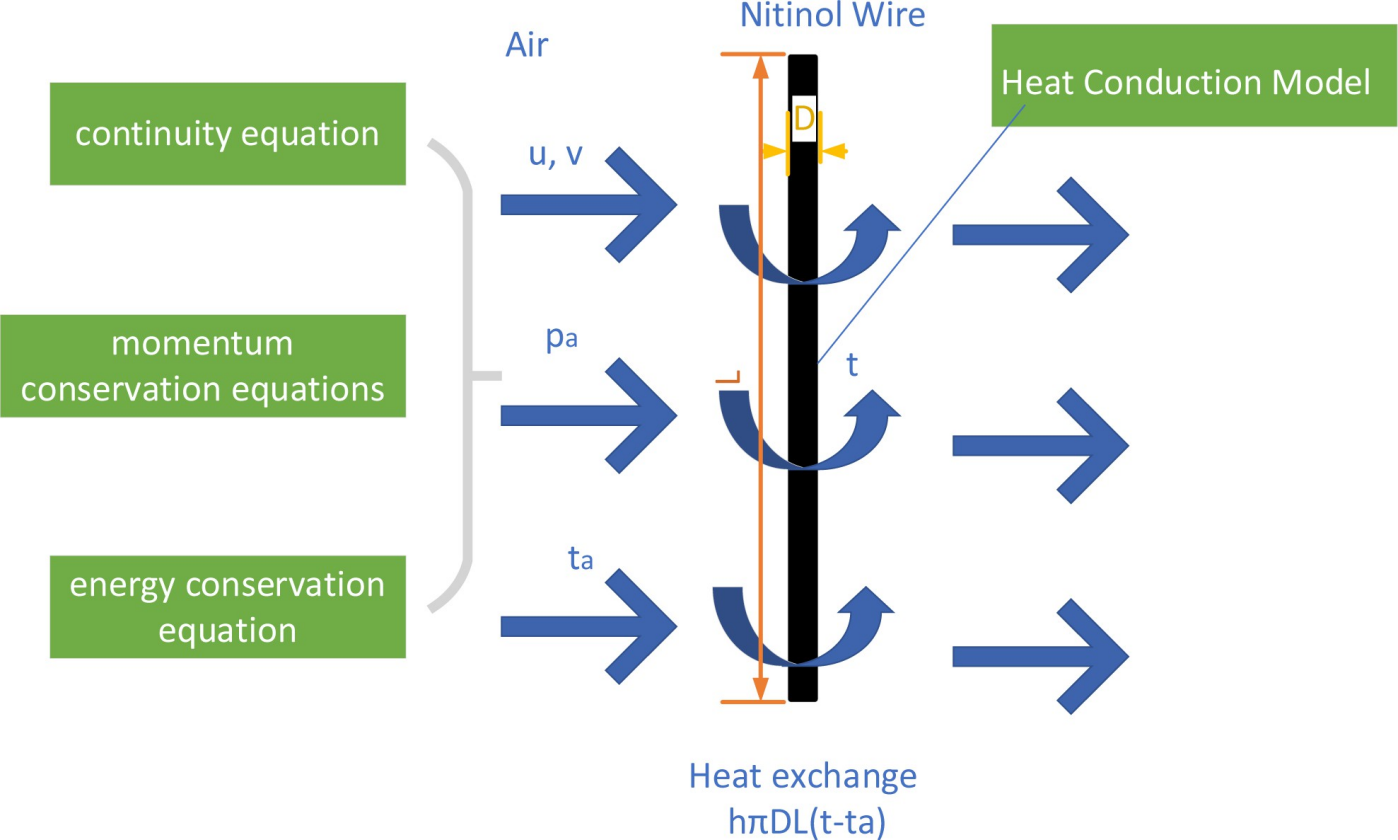
Sketch map of the coupled flow and convective heat transfer model.

A CFCHT model was built to predict the heating and cooling capacities of the wires in the nitinol wire–stepping motor unit and the module (Fig 5). Air was introduced for heat exchange with the wire. The airflow across the wire satisfies the continuity equation and the conservation of momentum and energy equations.

The continuity equation is expressed as

$$\frac{\partial u}{\partial x}+\frac{\partial v}{\partial y}=0.$$ (6)

The momentum conservation equations are expressed as

$$\rho_{a}\left(\frac{\partial u}{\partial \tau }+u\frac{\partial u}{\partial x}+v\frac{\partial u}{\partial y}\right)=F_{x}-\frac{\partial p}{\partial x}+\mu \left(\frac{\partial^{2}u}{\partial x^{2}}+\frac{\partial^{2}u}{\partial y^{2}}\right),$$ (7)

$$\rho_{a}\left(\frac{\partial v}{\partial \tau }+u\frac{\partial v}{\partial x}+v\frac{\partial v}{\partial y}\right)=F_{y}-\frac{\partial p}{\partial y}+\mu \left(\frac{\partial^{2}v}{\partial x^{2}}+\frac{\partial^{2}v}{\partial y^{2}}\right).$$ (8)

The energy conservation equation is expressed as

$$\frac{\partial t}{\partial \tau }+u\frac{\partial t}{\partial x}+v\frac{\partial t}{\partial y}=\frac{\lambda_{a}}{\rho_{a}c_{a}}\left(\frac{\partial^{2}t}{\partial x^{2}}+\frac{\partial^{2}t}{\partial y^{2}}\right),$$ (9)

where *ρ*_*a*_, *c*_*a*_, and *λ*_*a*_ are the density, specific heat capacity, and conductivity of the air, respectively; *u* and *v* are velocities along the *x* and *y* direction, respectively.

For the CFCHT model, Eqs (6–9) were used to calculate the velocity, temperature, and pressure distribution of air flowing across the nitinol wire. The velocity distribution was used to obtain the value of convective heat transfer coefficient *h* around the wire in Eq (3) to calculate the actual heating or cooling capacity as follows.

Heating capacity:

$$Q_{h}=h\pi DL(t-t_{a})=\int_{0}^{H}c_{a}m_{a}(t_{ao}-t_{a})dy$$ (10)

Cooling capacity:

$$Q_{0}=h\pi DL(t_{a}-t)=\int_{0}^{H}c_{a}m_{a}(t_{a}-t_{ao})dy$$ (11)

where *m*_*a*_ and *t*_*ao*_ are the mass flow per unit height and outlet temperature of air, respectively; *H* is height of the air passage.

### Heating and cooling capacities of the continuous torsional refrigeration module

The PHCC of a single nitinol wire or a combination of wires in the nitinol–stepping motor unit equals to *Q*_*v*_ if it is totally utilized during the continuous torsional process. Then, the PHCCs of the CTRS shown in Fig 1 can be estimated.

Set the stepping motor number to *2m*, the stepping motor speed to *n*_*1*_, and the servo motor speed to *n*_*2*_. In the cooling and heating areas, each stepping motor operates to drive the nitinol wires to twist *l* turns, and then stops during the following buffer time of *Δt*_*b*_. Therefore, the time for the wire to pass through each area in the stepping motor is

$$t_{1}=\frac{60l}{n_{1}}+{\Delta t}_{b}.$$ (12)

When the servo motor rotates once and the stepping motor rotates *2l* times, the corresponding servo motor speed is

$$n_{2}=30/t_{1}=n_{1}/2l.$$ (13)

Thus, the cooling capacity (*Q*_*t*0_) and heating capacity (*Q*_*th*_) of a continuous torsional refrigeration module are as follows:

$$Q_{t0}=\sum_{i=1}^{m}\frac{\int_{i\Delta t}^{(i+1)\Delta t}Q_{0}(i)dt}{\Delta t},$$ (14)

$$Q_{th}=\sum_{i=1}^{m}\frac{\int_{i\Delta t}^{(i+1)\Delta t}Q_{h}(i)dt}{\Delta t},$$ (15)

where the rotation time difference between two adjacent nitinol wires is

$$\Delta t=\frac{t_{1}-{\Delta t}_{b}}{m}.$$ (16)

### Grid independence verification

**Fig 6:**
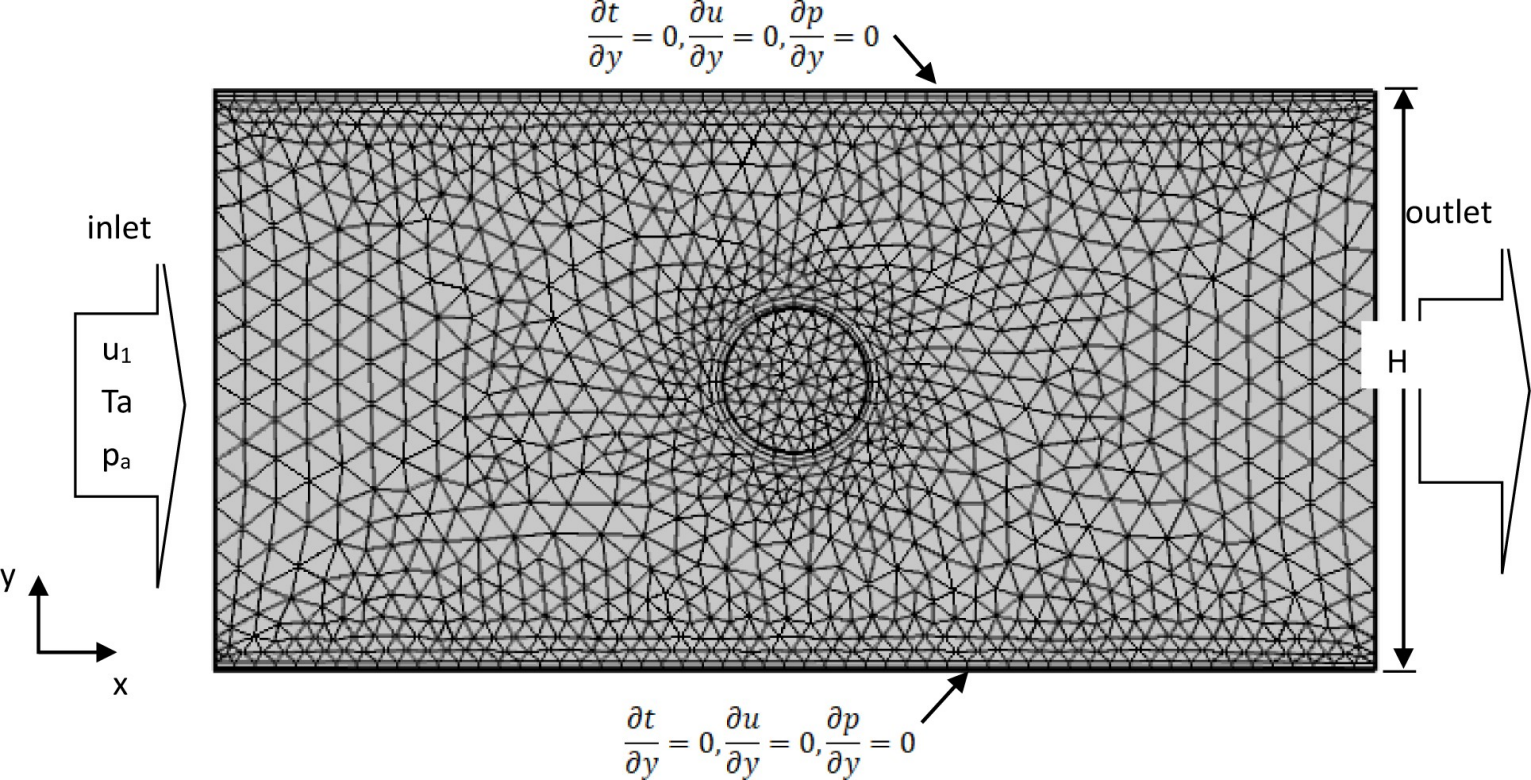
The overall grid schematic of the coupled flow and convective heat transfer model.

The equations were solved using engineering simulation software COMSOL MULTIPHYSICS 5.5. A calculation domain with a length of 5 and 10 cm parallel and perpendicular to the wire axis, respectively, was selected around the center of the wires. Coarser, coarse, normal, and fine meshes were calculated for this domain, as shown in Table 2. Since the deviations of these meshes were within ±5%, subsequent calculations were performed using a standard meshes to reduce the computation time. The domain was meshed with free triangular shapes and the near-field around the wire was refined along the boundary layer with a boundary stretching factor of 1.2. The maximum and minimum gird sizes were 0.268 and 0.012 mm, respectively; the spatial resolution in the near-field (the narrow regions in COMSOL) was 1; and the curvature factor was 0.3. Fig 6 shows a schematic of the grid in the model.

**Table 2:** Grid independent validation.

	Element number	Optimization objective	Solution time
Coarser	161	0.33672	491s
Coarse	533	0.32872	599s
Normal	1133	0.32672	784s
Fine	1933	0.32672	1139s

**Fig 7:**
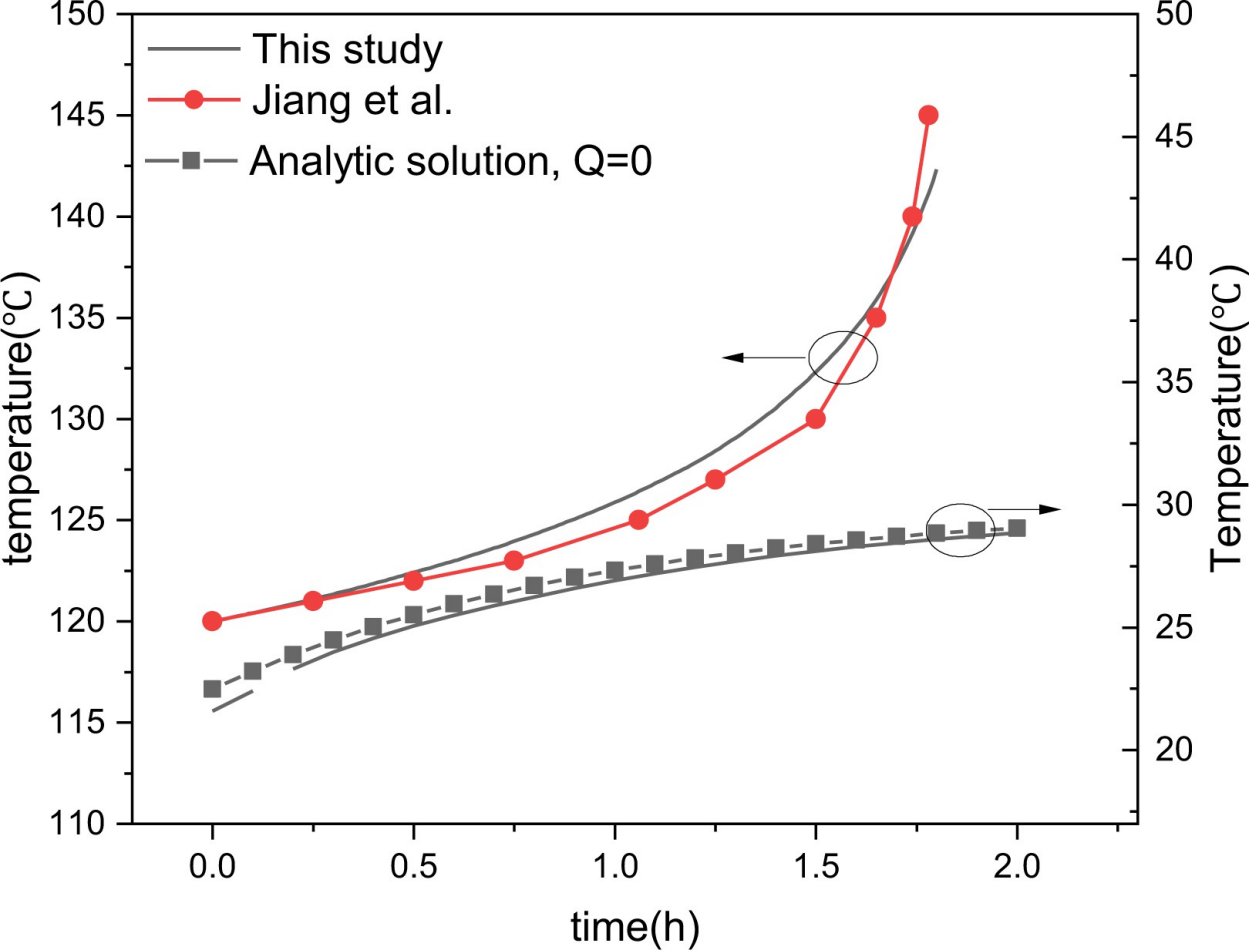
Verification of the coupled flow and convective heat transfer model.

The first set of data from Jiang et al. [19] and using the same properties and parameters was used to verify our model (Fig 7); the important values for this validation step were a maximum internal heat source of 0.5816MJ/kg, a thermal conductivity of 0.1108W/m·K, a density of 1111kg/m^3^, a heat capacity at constant pressure of 1582.6J/kg·K, and an initial temperature of 120°C. The second set of data comprises the analytic solution of the 1D unsteady heat conduction in the cylinder [17]; the important values for the second validation step were a zero internal heat source, a thermal conductivity of 34.8W/m·K, a density of 7800kg/m^3^, a heat capacity at constant pressure of 642J/kg·K, and an initial temperature of 200°C. It can be seen in Fig 7 that the model shows good agreement with both reference datasets with an average relative deviation between them of just 4%. Thus, the present model can accurately simulate the time history of the cylinder temperature.

## Experimental test

### Materials

The material used was a single nitinol wire with a nickel content of 56%, a titanium content of 34%, a diameter of 0.6 mm, and a test length of 50 cm.

### Experimental schemes

The number of alloy wires and the speed of the stepping motor were varied to test the heat absorption and release characteristics of the continuously twisted nitinol wires using the test rig. The test schemes are as follows:

1. One stepping motor was installed on the outer wheel. One end of the nitinol wire was connected to the stepping motor shaft, while the other end was connected to the outer wheel. The speed of the stepping motor was set to 50 rpm, and the stepping motor was switched on to begin twisting the nitinol wire 40 turns; this was the loading process. The stepping motor then reversed to make the wire untwist 40 turns back to the original state; this was the unloading process. The above loading and unloading processes were repeated for the continuous torsional experiment.
2. To obtain two-twisted-wire combinations, two alloy wires were selected and twisted together clockwise in advance. Thereafter, one end of each wire combination was fixed to the stepping motor shaft and the other end to the outer wheel, and the two-twisted-wire combinations were tested using Step 1.
3. The experimental schemes for the variable speed torsion test are as follows:
4. The two-twisted-wire combination obtained using the procedure described in Step 2 was installed on the outer wheel and the stepping motor. The stepping motor speed was set to 45 rpm, the stepping motor was switched on, and the test was performed as described in Step 1.
5. The stepping motor speed was set to 40 rpm, the stepping motor switched on, and the test was performed using Step 1.

### Data processing

During the experiment, the temperature of the nitinol wires was continuously measured by an infrared thermal imager (Testo 8852) calibrated and validated by the standard thermometer, and the temperature at the midpoint (potin A in Fig 1) of the wires was measured to analyze their thermal characteristics.

## Results and discussions

### Temperature

**Fig 8:**
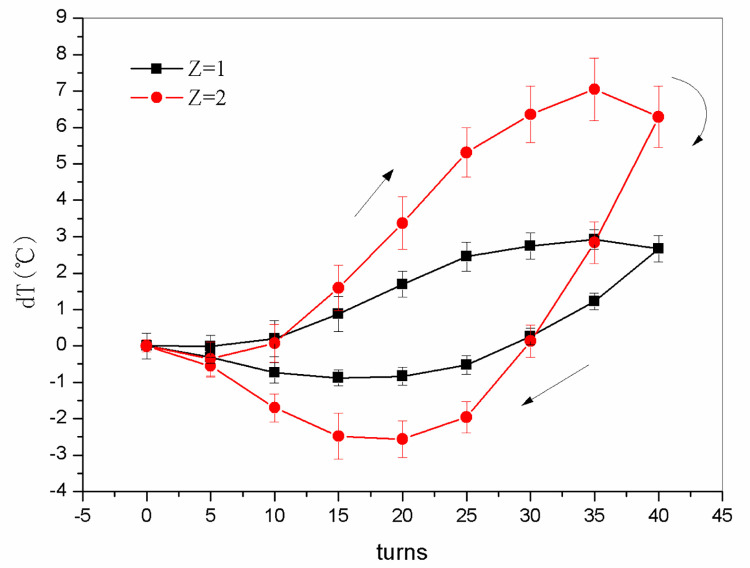
The temperature difference between the nitinol wire surface and the ambient air of the CTRS (*dT*) as a function of the number of alloy wires (*Z*) and twist turns.

Fig 8 shows the change in temperature difference between the surface of a single nitinol wire in the nitinol wire-stepping motor unit and ambient air, as well as that between the two-twisted-wire combination in the nitinol wire-stepping motor unit and ambient air, during the continuous alternate torsional process. As the stepping motor rotated the nitinol wire clockwise, the temperature of the wire increased before 30–35 turns. Thereafter, the temperature decreased slightly but remained higher than the ambient temperature owing to the deceleration of the stepping motor and the increase in heat transfer between the wire and the environment. As the motor rotated counterclockwise (unloading), the temperature of the nitinol wire surface decreased before unloading to 10–20 turns. The temperature then gradually increased but remained lower than the ambient temperature. This variation with temperature is in agreement with the findings of Ossmer et al. [20].

As the stepping motor operated, the stress on the nitinol wire increased and the austenite-to-martensite phase transformation occurred when the stress surpassed the phase change saturation stress. During the process, entropy decreased, and latent heat was released into the ambient air. The reverse martensite-to-austenite phase transformation occurred when the stepping motor operated in reverse, thereby decreasing the stress to below the saturation stress of the phase change. The entropy increased, and the nitinol wire absorbed heat from the ambient air, which produced the cooling effect [21].

After the loading process began, the temperature gradually increased. This incremental behavior differs from that of strip materials that only experience a moderate increase in stress before the temperature begins to change [20]. This is mainly because the critical transformation pressure required for martensitic transformation is easy to achieve in the torsional wire driven by the motor [22]. The temperature change that occurs during the unloading process is similar to that of strip materials, in that once unloading begins, the temperature decrease.

The temperature change in nitinol wires during torsion infers that they can be used in a CTRS to provide continuous heating and cooling while passing through the heating and cooling zones, respectively. During the loading stage, the nitinol wires release heat, which can be absorbed by air, water, or other fluids to produce heating while the nitinol wires are cooled. During the unloading stage, the nitinol wires absorb heat from the fluid flowing around them, which reduces the temperature of the fluid and subsequently provides refrigeration. Notably, the temperature of the nitinol wires in the final stage in the heating zone has a direct impact on the refrigeration effect in the cooling zone. The refrigeration effect improves as the temperature of the nitinol wires decreases.

As shown in Fig 8, the maximum temperature rise and drop between one nitinol wire and ambient air during loading and unloading were 1.4 and 0.6°C, respectively, and between the two nitinol wires and ambient air were 7.1 and 2.6°C, respectively. Thus, twisting and untwisting two wires together produced a significantly higher temperature change than with a single wire. Increasing the number of transformable materials can increase the temperature change in SMAs induced by twisting and untwisting [13], while the heat absorbed and released by twisting and untwisting two nitinol wires were significantly higher than with one wire because of the geometric dimension enlargement. Moreover, Xu et al. [15] discovered that a rectangular section of the martensite phase with a larger geometric size has a higher average temperature change rate than a trapezoidal section. Since the effect with two wires is enhanced, the following test was performed for two-wire combinations in one nitinol wire-stepping motor unit.

Based on the temperature change in the wire relative to the ambient temperature, the temperature rise during loading was greater than that during unloading, which is the same as with torsional strip materials [23]. This trend can be attributed to friction or other diffusion mechanisms [24]. The measured temperature changes for stretched strip materials are larger (22.5 and 17.0 K for loading and unloading, respectively) [20]. The temperature variation in the present work was smaller because the section area of the wire was smaller than that of strip materials.

**Fig 9:**
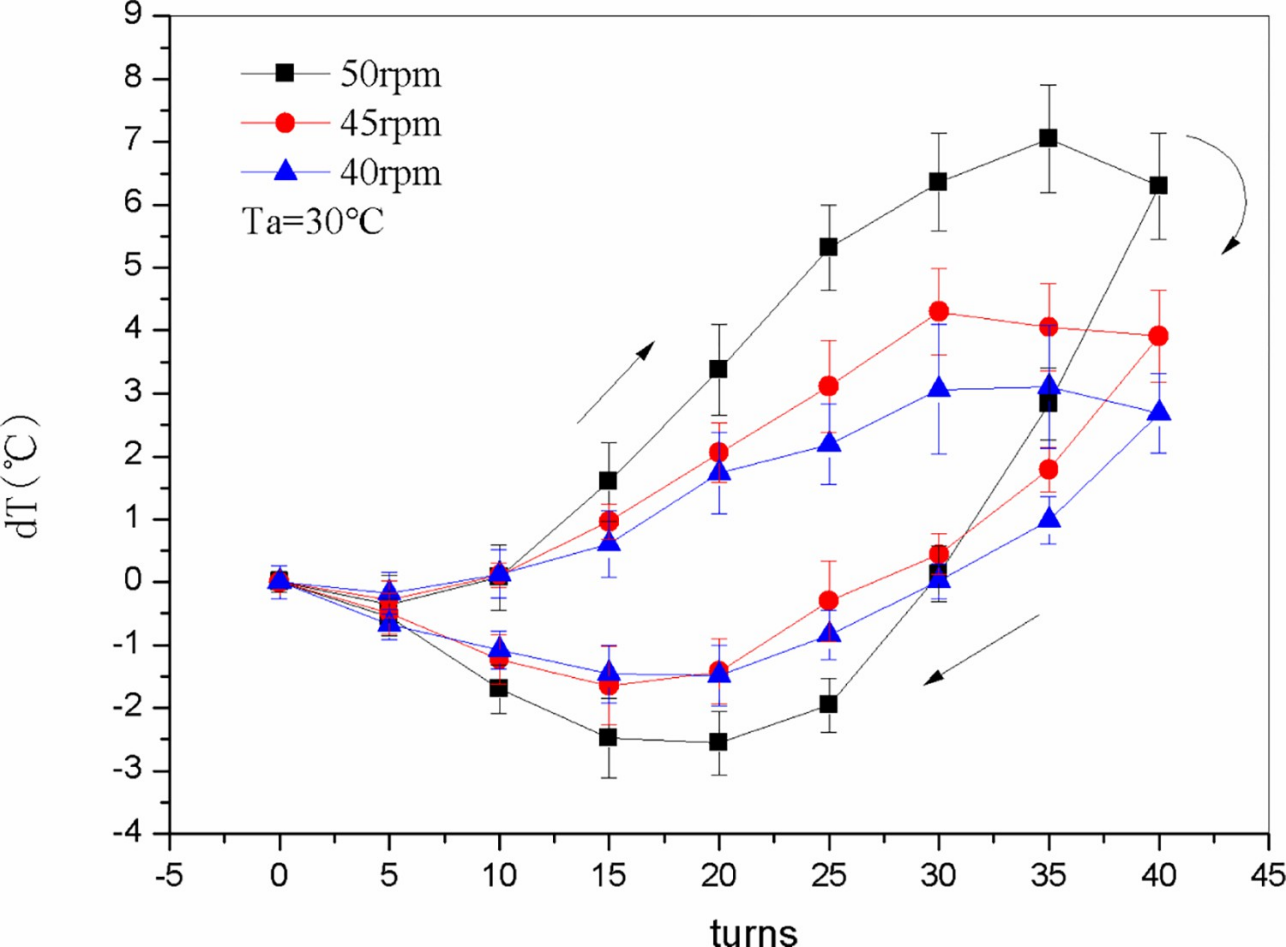
Temperature difference between the surface of a two-nitinol-wire combination and ambient air (*dT*) as a function of the stepping motor speed (*n*_*1*_).

Fig 9 shows the difference between the wire surface temperature and the ambient temperature when the stepping motor rotated the wire at various speeds. It can be seen that when the stepping motor speed was increased from 40 to 50 rpm, the maximum temperature rise of the nitinol wire relative to ambient temperature increased from 3.1 to 7.1°C (a 129% increase) while the maximum temperature drop increased from 1.5 to 2.5°C (a 66.7% increase), indicating that the temperature at the wire surface is related to the rotational speed: the faster the rotational speed, the greater the temperature change. The reason could be that higher speed results in a higher strain rate and the temperature change is directly related to the strain rate [20]. However, high speed requires high torque, which leads to increased power consumption and large driving motors, which makes the system uneconomical. Therefore, a comprehensive trade-off between motor speed and temperature change demand must be made in the design.

Based on the experimental results, the above optimization model and CFCHT model were used to calculate heat production, and cooling and heating capacities with the parameters reported in Table 3.

**Table 3:** Model parameters.

parameters	value
Diameter of the wire	0.6mm
length of the wire	0.5 m
ρ	6500 kg/m3 [20]
c	0.45 kJ/g·k [20]
λ	18W/mK [20]
Ɛ	0.9
Ta (initial temperature)	304.15K
h	Natural convection heat transfer correlation from [17]
Q_d0_	0.1(iterative initial value)
Q_a0_	-0.1(iterative initial value)
k_1_	0.2(iterative initial value)
k_2_	0.2(iterative initial value)
u_1_	0.5, 1.0, 1.5, 2.0m/s
pa	101325Pa
H	0.5m

### The heat production

The *Q*_*v*_ expression of the nitinol wires was obtained using the above optimization model, as shown in Table 4. The surface temperature-based determination coefficient was calculated using the following equation:

$$R^{2}=1-\frac{\sum_{i=1}^{m}{\left(t_{ci}-t_{ei}\right)}^{2}}{\sum_{i=1}^{m}{\left(t_{ei}-\bar{t_{e}}\right)}^{2}}.$$ (17)

**Table 4:** Coefficients of heat production expression and determination coefficients of the nitinol wire surface temperature.

		Parameters in Eqs (4–5)				R^2^
		Q_d0_	k_1_	Q_a0_	k_2_	
Number of alloy wires	1	0.2225	0.0962	−0.26078	0.10914	0.963
	2	0.32272	0.05536	−0.32242	0.11042	0.945
speed of stepping motor	50 rpm	0.65705	0.0542	−0.64709	0.1046	0.959
	45 rpm	0.41815	0.06224	−0.40541	0.1102	0.943
	40 rpm	0.27065	0.084937	−0.38556	0.13072	0.943

Table 4 shows that the nitinol wires generated and absorbed heat during loading and unloading, respectively. The heat production changed with the number of nitinol wires and the motor speed. The temperature distribution of each point of the nitinol wire can be obtained by substituting the optimized heat product value into the conduction model. The determination coefficient between the simulated and experimental surface temperature values was more than 0.9, indicating that the model is rational.

### The potential heating and cooling capacities of a nitinol wire-stepping motor unit

**Fig 10:**
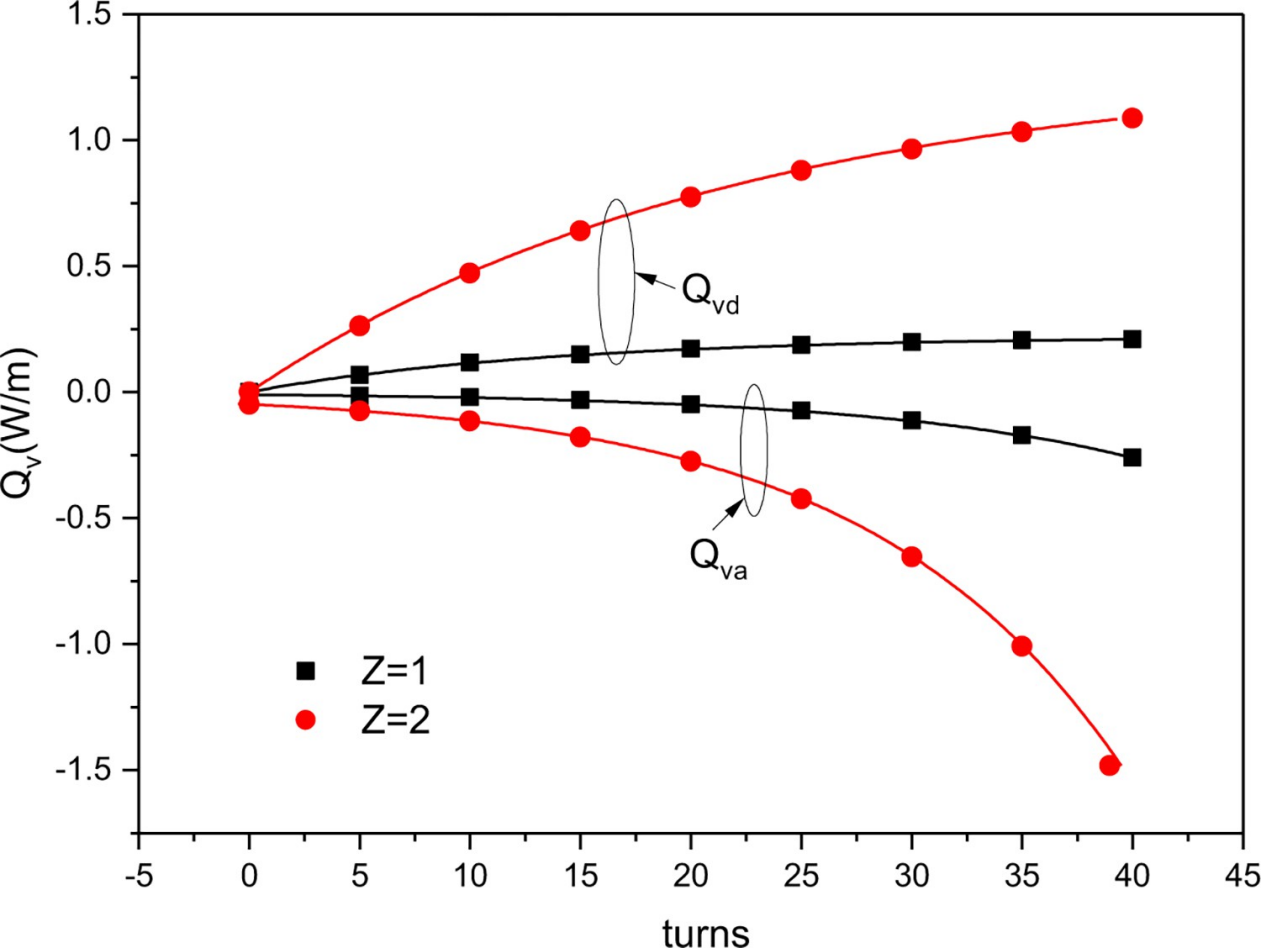
Changes in simulated PHCCs with the number (*Z*) and twist turns of the nitinol wires in one nitinol wire-stepping motor unit during continuous torsional refrigeration.

Fig 10 illustrates how the number and twists of the nitinol wire in one nitinol wire-stepping motor unit affected the PHCCs during continuous torsional refrigeration. It can be seen that when the wire was loaded, heat was released, and the amount gradually increased as the torsion progressed. When the wire was unloaded, it absorbed heat, with the largest heat absorption occurring at the beginning. As the unloading process progressed, the amount of heat absorbed gradually decreased until it reached zero. Fig 10 also shows that the entanglement of the two nitinol wires increased the torsion stress and increased the maximum heat release from 0.21 to 1.09 W/m and the maximum heat absorption from 0.26 to 1.48 W/m. Thus, the heat dissipation and absorption increased as the number of torsion wires was increased. According to the maximum heat release and absorption data, the heat absorption effect of the wire during the continuous torsional process is greater than its heat release effect, implying that the refrigeration effect is predominant over the heating effect.

**Fig 11:**
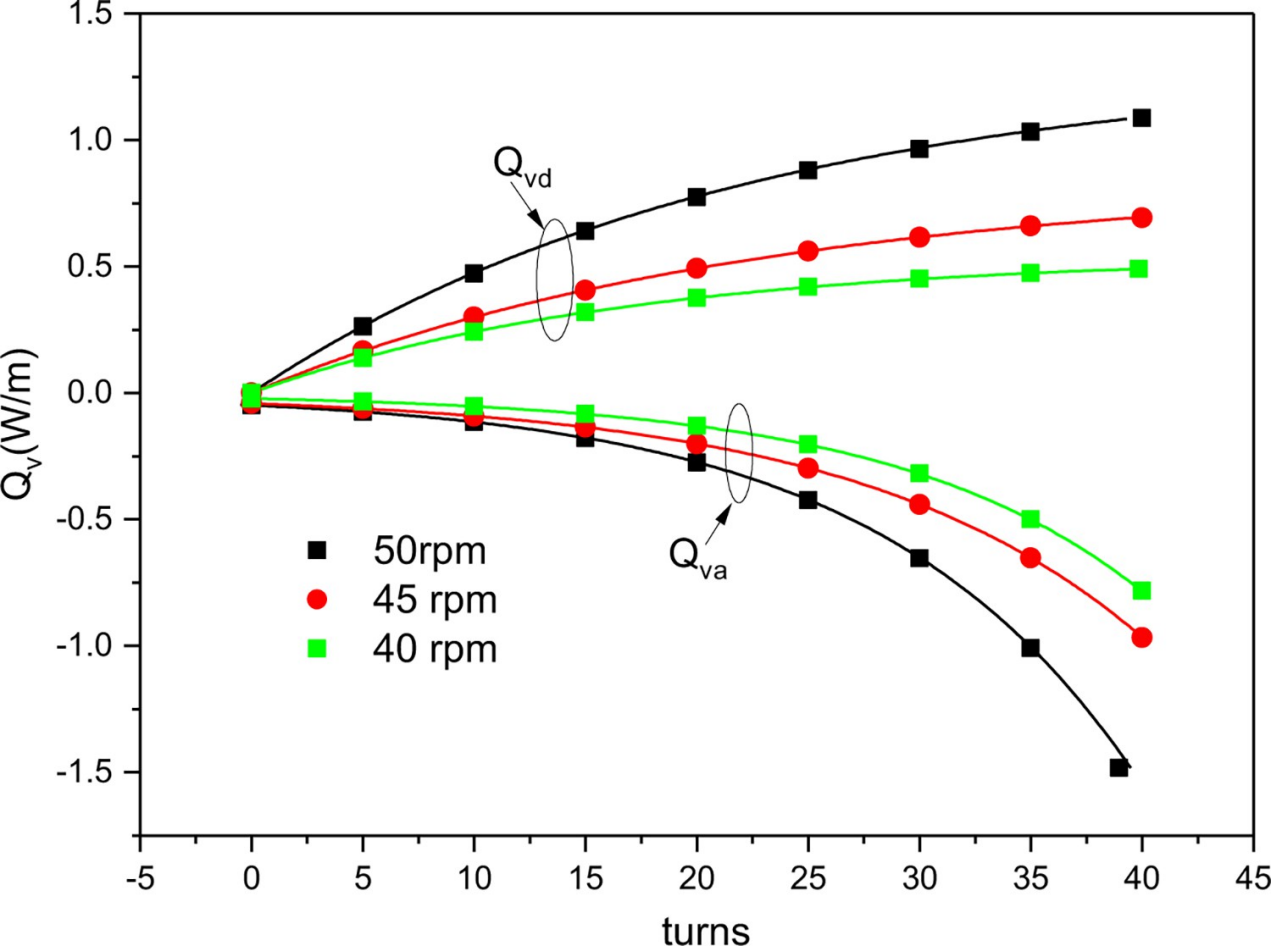
Simulated PHCCs as a function of the stepping motor speed (*n*_*1*_).

Fig 11 shows how the PHCCs of using two nitinol wires in the unit changed with the number of torsional turns and the speed of the stepping motor during continuous torsional refrigeration. The variation in heat release and absorption with the number of the torsional turns during the loading and unloading processes is consistent with Fig 10. During the loading process, as the number of torsional turns increased, the martensite phase gradually transformed to the austenite phase [25], thereby increasing the austenite composition in the nitinol wire and releasing heat. During the unloading process, the martensite-to-austenite phase transformation occurred rapidly in the nitinol wire, with the largest heat absorption occurring at the beginning and then gradually decreasing to zero. This means that the latent heat from the phase transformation in the wire is continuously released or absorbed as the crystal state changes under the influence of an external force. Fig 11 also shows that after the stepping motor speed was decreased from 50 to 45 rpm, the maximum heat release decreased from 1.09 to 0.69 W/m and the maximum heat absorption decreased from 1.48 to 0.81 W/m. When the stepping motor speed was decreased to 40 rpm, the maximum heat release decreased to 0.49 W/m and the maximum heat absorption decreased to 0.78W/m, which are 0.2 and 0.03 W/m lower than the maximum heat release and heat absorption at 45 rpm, respectively. Thus we can conclude that the phase transition in the nitinol wire decelerates with a decrease in the stepping motor speed, resulting in relatively low heat release and absorption.

### Cooling and heating capacities

#### The cooling and heating capacities of a nitinol wire-stepping motor unit

**Fig 12:**
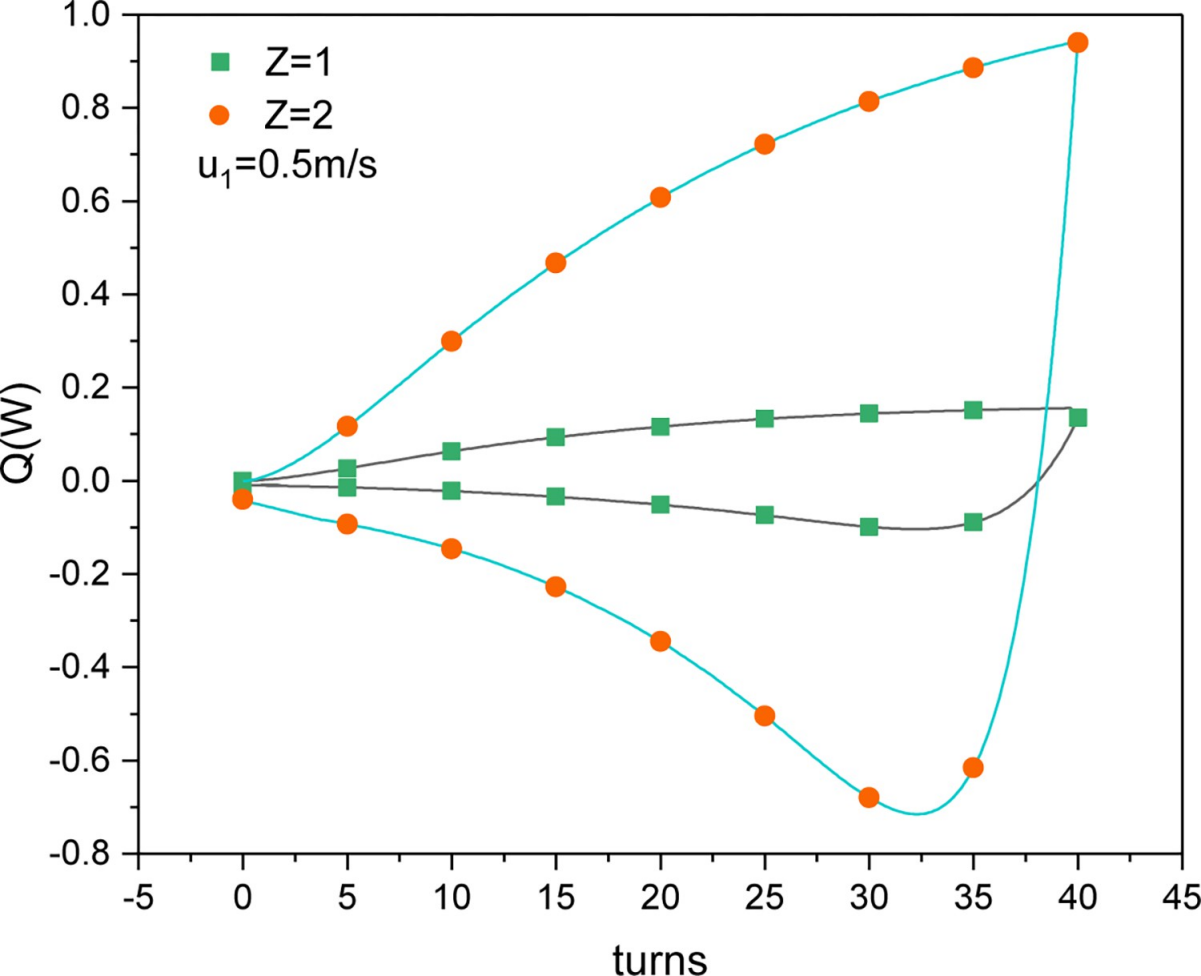
Changes in the simulated cooling and heating capacities of a nitinol wire-stepping motor unit.

In general, the heat per unit mass or volume of the phase change latent heat is used to evaluate the performance of modern elastic refrigeration systems [26]. However, the actual engineering application is focused on the cooling capacity per unit of time. Fig 12 shows the effect of the number of nitinol wires and the number of turns on the cooling and heating capacities of nitinol wire-stepping motor units with either one or two nitinol wires. As shown in Fig 12, the heating capacity increased as the number of torsional turns increased during the loading process. After switching from loading to unloading, the endothermic phase change caused the air outlet temperature to decrease from the maximum value reached at the end of the heating stage, indicating that the heating effect continued until the outlet air temperature was lower than the ambient temperature. Thereafter, the cooling capacity gradually decreased as the unloading progressed. Thus, the nitinol wire can cool down and heat up during continuous torsion. The maximum heating capacities with one and two nitinol wires in the nitinol wire-stepping motor unit were 0.14 and 0.94 W, respectively, while the maximum cooling capacities were 0.1 and 0.72 W, respectively. Thus, the heating and cooling effects of using two nitinol wires in the nitinol wire-stepping motor unit were enhanced compared to using one wire, just as was seen with heat production.

**Fig 13:**
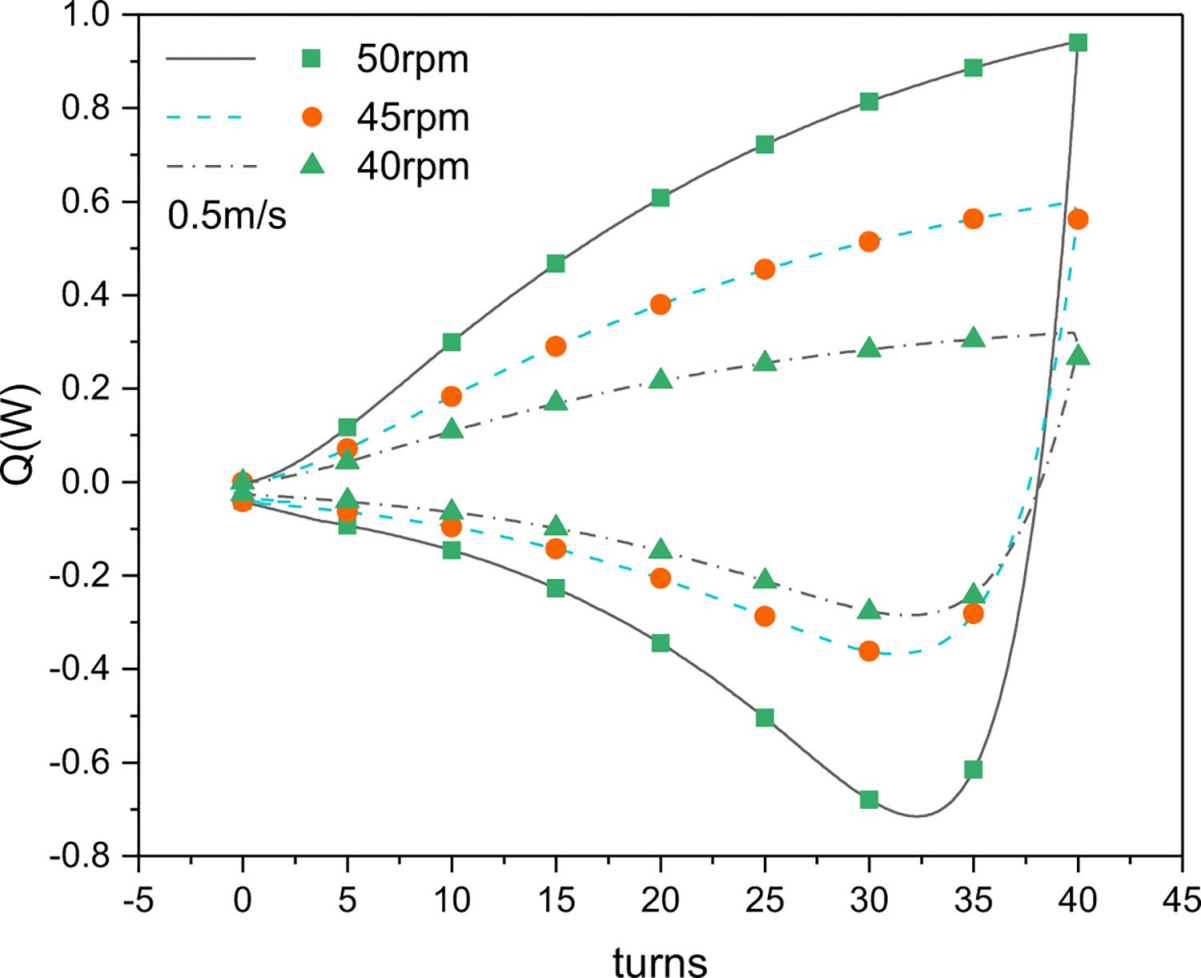
Simulated cooling and heating capacities of a single nitinol wire–stepping motor unit as functions of the stepping motor speed (*n*_*1*_).

As shown in Fig 13, the cooling and heating capacities of one nitinol wire-stepping motor unit with two twisted wires changed with the rotational speed of the stepping motor. When the stepping motor speed was increased from 40 to 50 rpm, the maximum heating capacity increased from 0.319 to 0.94 W and the maximum cooling capacity increased from 0.284 to 0.72 W. Hence, the available heating and cooling capacities were lower than the heat generation and absorption by the wires, respectively, because of the existence of thermal inertia factors.

**Fig 14:**
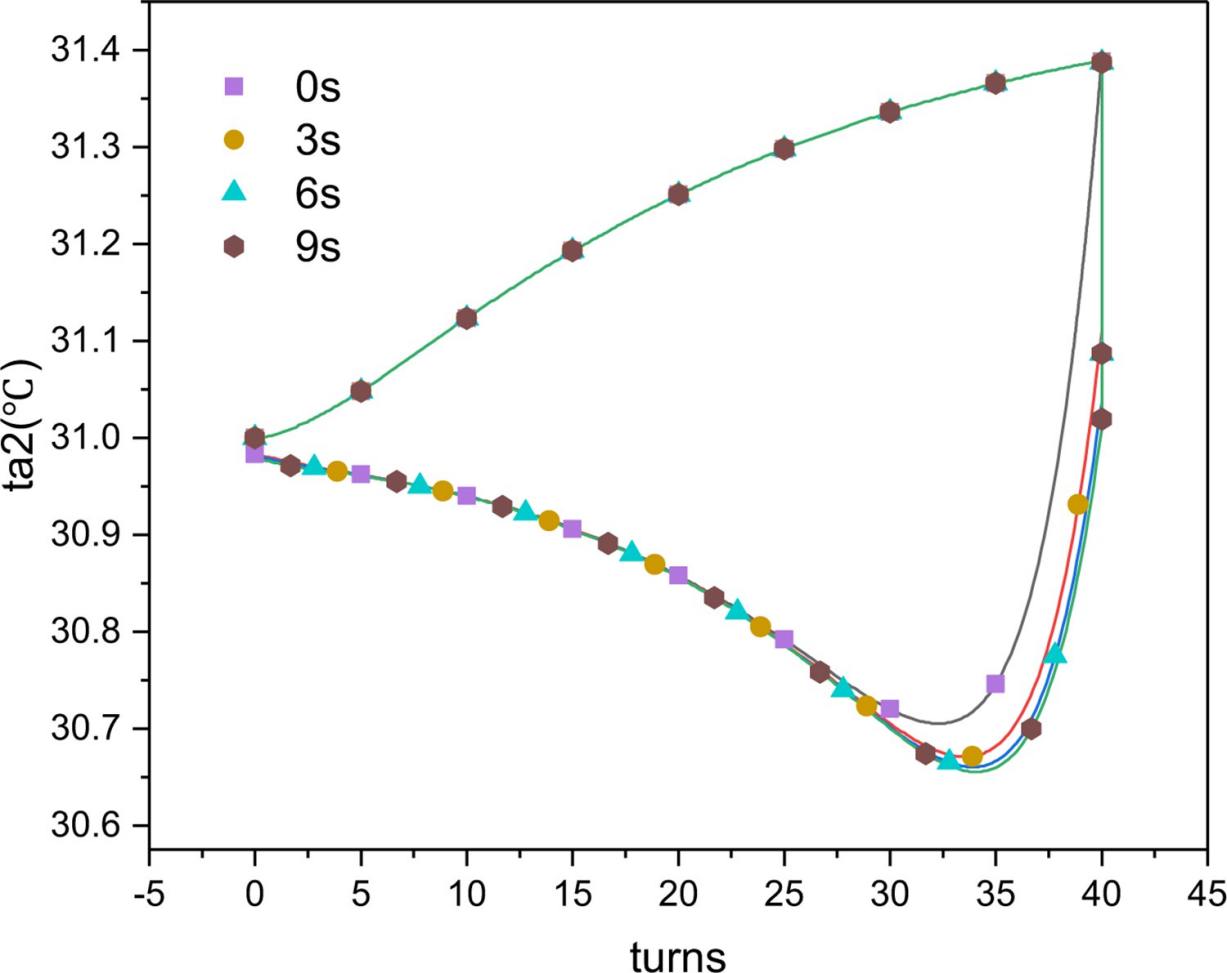
Changes in the simulated air outlet temperature with buffering time during continuous torsion of the nitinol wires (*u* = 0.5 m/s, *n*_*1*_ = 50rpm).

**Fig 15:**
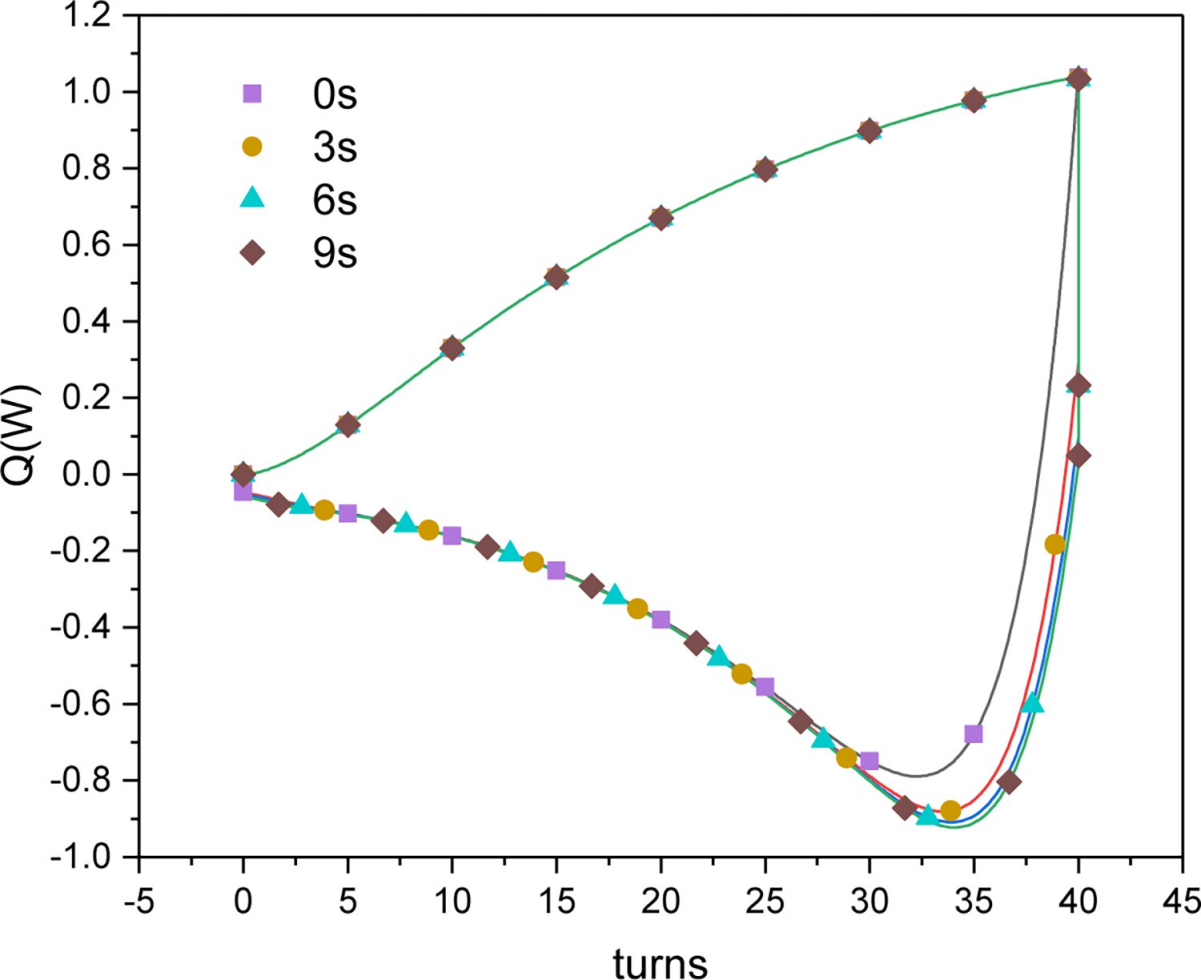
Simulated cooling and heating capacities of the nitinol wires in a continuous torsional refrigeration unit as functions of buffering time (*u* = 0.5 m/s, *n*_*1*_ = 50rpm).

As shown in Figs 12 and 13, when the wires were directly switched from loading to unloading mode, the air outlet temperature could not immediately decrease to below the ambient temperature to cool the inflow air. Therefore, when the loading mode ended, the stepping motor first underwent buffering, during which the surface temperature of the wire reached the ambient temperature under the cooling of the inflow air. Thereafter, the unloading mode started, and the ambient temperature decreased under heat absorption, resulting in effective refrigeration with reduced cooling and heating offsets during the continuous torsional process. Figs 14 and 15 show changes in the air outlet temperature and the cooling and heating capacities of the nitinol wires with and without buffering, respectively. It can be seen that without buffering, the unloading process started immediately after the loading process had completed. At the end of the loading process, the air outlet temperature reached 31.4°C and then started falling because of the simultaneous endothermic phase change and ambient air cooling. The desired refrigeration can only be achieved when the outlet temperature is lower than the ambient temperature. When the buffering was set to 3, 6, or 9 s, the unloading process did not start immediately after the wires had been loaded. Instead, the rotation of the stepping motor was temporarily halted, and the temperature of the wires was reduced via air cooling. After buffering had completed, the unloading process began and the phase-transformed heat absorption by the wires caused the outlet temperature to decrease rapidly. Buffering times of 3, 6, and 9 s increased the refrigerating capacity to 0.88, 0.91, and 0.92 W, respectively. Although a relatively long buffering time increased the refrigerating capacity, it reduced the torsional time and affected the heating output of the whole device. Therefore, it should not be set to be too long. When the buffering time was 6 s, the outlet temperature decreased to the ambient temperature at the end of the buffering period. Thus, a buffering time of 6 s was suitable and was used in the subsequent analyses.

**Fig 16:**
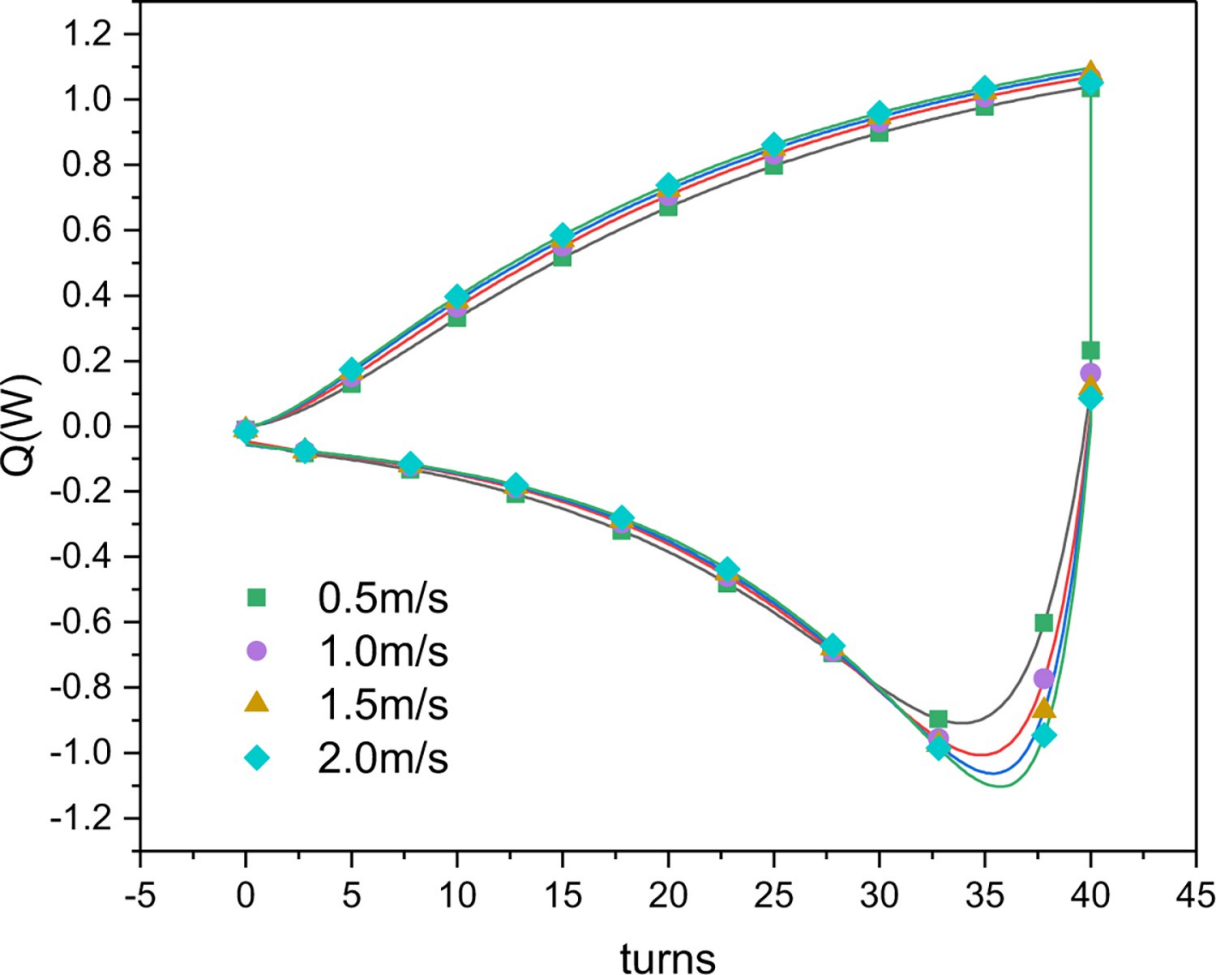
Simulated cooling and heating capacities of the nitinol wires in a nitinol wire-stepping motor unit as functions of airflow rates (*n*_*1*_ = 50rpm).

Fig 16 shows how the cooling and heating capacities of the nitinol wires in a single nitinol wire-stepping motor unit changed with the airflow rate. It can be seen that the maximum cooling and heating capacities increased as the airflow rate increased. When the airflow rate increased from 0.5 to 2.0 m/s, the maximum cooling capacity increased from 0.71 to 1.08 W, and the maximum heating capacity increased from 0.93 to 1.16 W.

**Fig 17:**
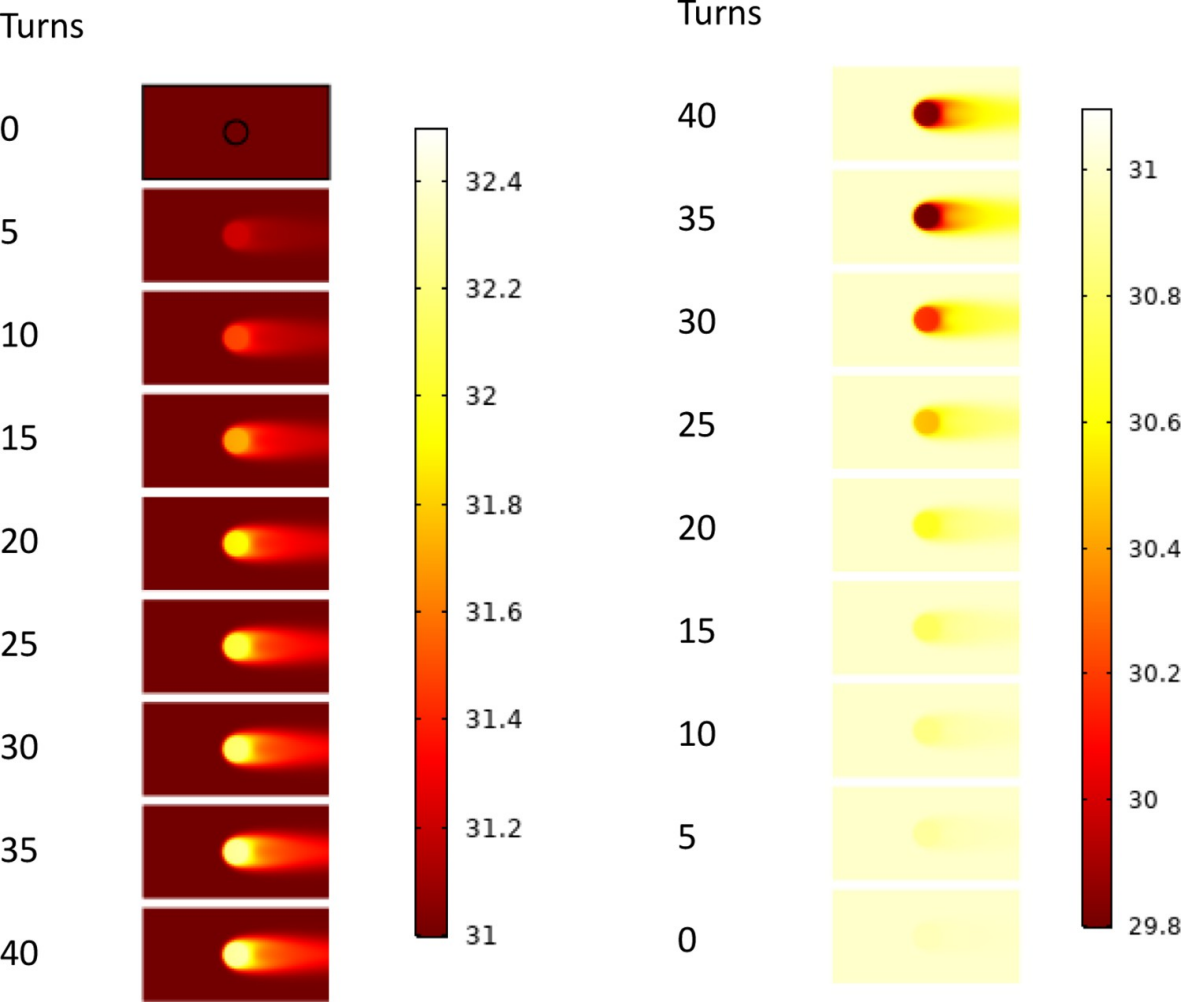
Simulated temperature distribution of the nitinol wires in a nitinol wire-stepping motor unit at various torsion turns (*n*_*1*_ = 50rpm).

Fig 17 shows the temperature distribution of the nitinol wires in a nitinol wire-stepping motor unit at various torsional turns. As can be observed, the air temperature gradually increased after loading because of the heat released by the torsional phase transformation. As the loading process progressed, the amount of heat released owing to phase change gradually increased and the air temperature gradually increased, while the maximum temperature difference between the inlet and outlet reached 1.4°C. During the unloading process, due to the heat absorbed by the phase change, the temperature of the nitinol wires decreased and absorbed the heat from the surrounding air, resulting in a decrease in the outlet air temperature and a maximum temperature difference of 1.2°C. However, as the unloading process progressed, the heat absorption due to the phase change decreased gradually, the temperature difference between the air inlet and outlet decreased, and the outlet air temperature increased gradually.

#### The cooling and heating capacities of the continuous torsional module

**Fig 18:**
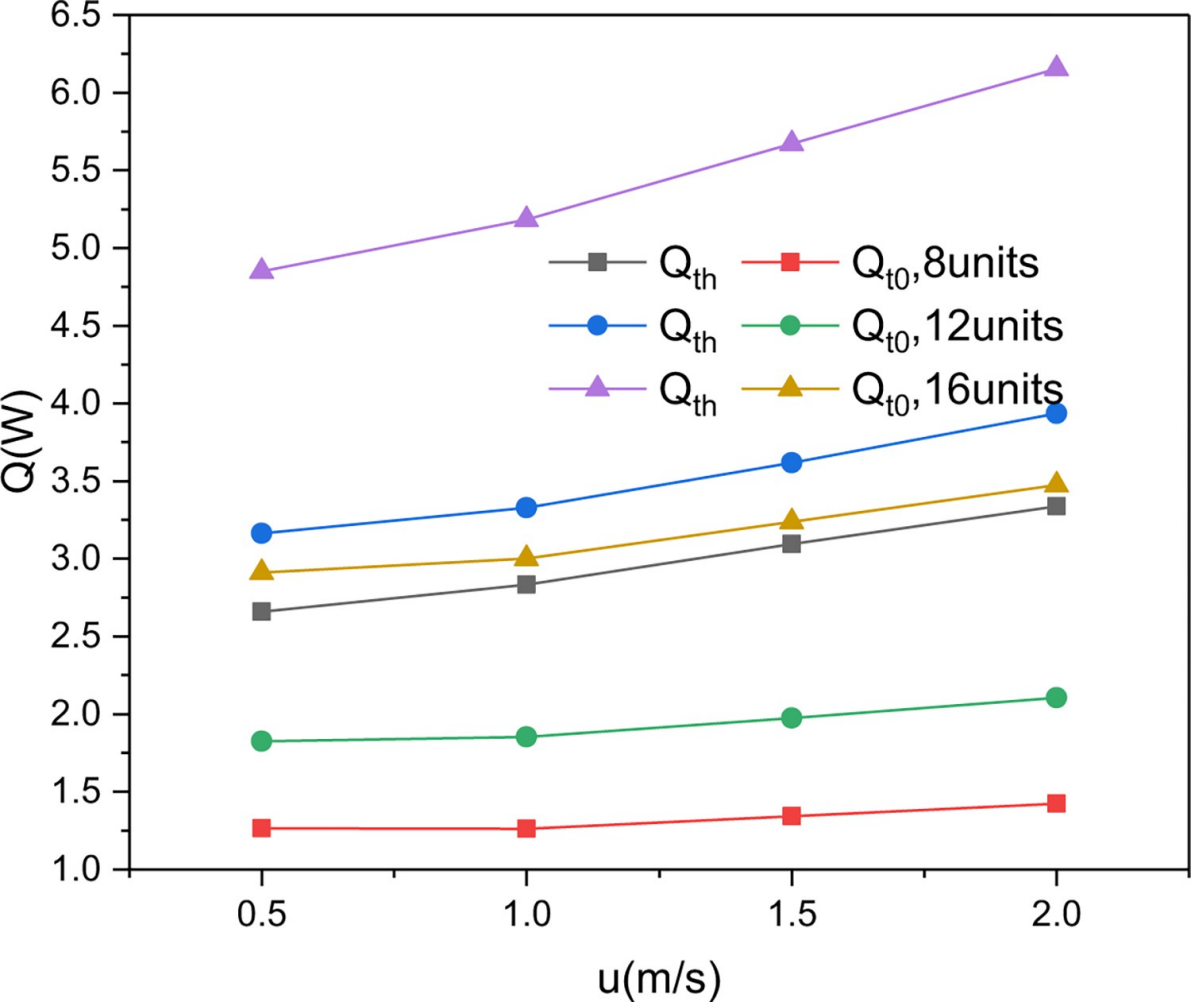
Simulated heating and cooling capacities of the continuous torsional refrigeration module as functions of the air velocity and alloy–stepping motor units.

The cooling and heating capacities of the nitinol wires shown in Fig 16 can be integrated using Eqs (14–15) to attain the total cooling and heating capacities of the continuous torsional refrigeration module (Fig 1), as shown in Fig 18. In the calculation, the rotational speed of the stepping motor *n*_*1*_ = 50 rpm and the number of nitinol wire-stepping motor units (*m*) was set to 8, 12, or 16.

Fig 18 shows how the cooling and heating capacities of the continuous torsional refrigeration module changed with the number of nitinol wire-stepping motor units and air velocity when the rotational speed of the stepping motor was 50 rpm. It can be seen that the heating and cooling capacities of the module increased as the air flow rate and the number of nitinol wire-stepping motor units was increased. For example, as the airflow rate was increased from 0.5 to 2.0 m/s, the heating and cooling capacities increased from 2.66 to 3.33 W and 1.26 to 1.42 W with 8 units; from 3.16 to 3.94 W and 1.82 to 2.11 W with 12 units, and from 4.85 to 6.15 W and 2.91 to 3.48 W with 16 units, respectively. Thus, higher heating and cooling capacities can be achieved by increasing the number of alloy wire-stepping motor units or using multiple modules in series.

The continuous torsional refrigeration module allows the nitinol wires to be loaded and unloaded continuously. Similar to the heat flow inside alloy wires during heat treatment [2, 27], the heat flow in the nitinol wires used in the present study was essentially due to the heat generated by the transformation between the austenite and martensite phases. The unique design of the continuous torsional refrigeration module enables it to perform cooling and heating either separately or simultaneously.

The specific cooling power (SCP; W/g) and the cooling/heating capacity are two criteria used to evaluate the refrigeration capacity in modern torsional refrigeration and heat pump research. SCP is a suitable index for determining the cooling power density per unit mass of SMAs [26]. The SCP value is dependent on the SMA type and size, device type, and operating mechanism. The SCP of the device investigated in the present study was 0.25–0.28 W/g with a rotational speed of the stepping motor of 50 rpm and two-nitinol-wire combinations, whereas the SCP of a single-stage elastic cooling system with an active elastic regenerator is 0–2 W/g [26]. Moreover, an active caloric regenerative (ACR) heat pump cycle has a heating capacity of 280–600 W [28, 29] with a heating SCP of 7.2–15.4 W/g, which is much higher than in the present study (0.53–0.66 W/g). The reason for the large difference is that, in addition to the different system forms, the ACR heat pump uses water as the heat exchange fluid to achieve forced convective heat transfer, which is more efficient than air used in the present study because it has better transfer characteristics in terms of physical parameters such as specific heat capacity, thermal conductivity, and density.

## Conclusions

A novel modular continuous torsional refrigeration test rig using twisted nitinol wires was built and experimental and simulation studies were conducted to determine the thermal characteristics, and PHCCs of the nitinol wires during the continuously alternating torsional process. The main conclusions were as follows:

1. The surface temperature increased as the stepping motor twisted the nitinol wires clockwise and decreased as it untwisted them counterclockwise. The thermal characteristics of the wires being twist and untwisted provided a valuable reference point for the zoning of the continuous torsional process and the development of a continuous torsional refrigeration prototype.
2. During the continuous twisting and untwisting of the nitinol wires with a stepping motor, the number of torsional turns and the number of wires significantly influenced the temperature of the nitinol wires. Two nitinol wires being twisted can be used for refrigeration and heating in the actual design of a torsional refrigeration system.
3. The measured temperature variation was used to optimize the PHCCs of the nitinol wires via the optimization model. Subsequently, a CFCHT model was used to predict the cooling and heating capacities. The simulation results reveal that the cooling and heating capacities of the continuous torsional refrigeration device were increased with the number of nitinol wires, the stepping motor speed, and the air velocity. Therefore, the capacity of a module can be improved by changing the number and geometric size of the wires, the number of torsional turns, and the stepping motor speed. Our approach could be valuable for research into caloric materials for torsional refrigerators with air as the medium. Our subsequent work for CTRS will include selecting materials with a higher twistocaloric effect, improving the module compactness, increasing capacity, and reducing energy consumption, thereby realizing this approach for making the next-generation domestic, commercial, and industrial refrigerators.
